# GATA3 Inhibits the Expression of Viral E6/E7 Genes, and Its Expression Is Compromised During HPV‐Mediated Cervical Carcinogenesis

**DOI:** 10.1002/jmv.71034

**Published:** 2026-06-26

**Authors:** Valéria Talpe‐Nunes, Aline Lopes Ribeiro, Rafaella Almeida Nunes, João Simão Sobrinho, Amanda Schiersner Caodaglio, Rossana Veronica Mendonza Lopez, Thais Rocha, Maria Luiza Nogueira Dias Genta, Konstanze Schichl, Ademola Aiyenuro, John Doorbar, Laura Sichero

**Affiliations:** ^1^ Laboratório de Biologia Molecular Instituto do Cancer do Estado de Sao Paulo, Hospital das Clínicas HCFMUSP, Faculdade de Medicina, Universidade de Sao Paulo São Paulo São Paulo Brazil; ^2^ Comprehensive Center for Precision Oncology, Instituto do Cancer do Estado de Sao Paulo, Hospital das Clínicas HCFMUSP, Faculdade de Medicina, Universidade de Sao Paulo São Paulo São Paulo Brazil; ^3^ Departamento de Patologia do Hospital das Clinicas HC FMUSP Hospital das Clínicas HCFMUSP Faculdade de Medicina, Universidade de Sao Paulo São Paulo São Paulo Brazil; ^4^ Hospital das Clínicas HCFMUSP, Faculdade de Medicina, Universidade de Sao Paulo São Paulo São Paulo Brazil; ^5^ Department of Pathology University of Cambridge Cambridge UK

**Keywords:** cervical cancer, GATA3, HPV, prognostic biomarker, transcription

## Abstract

High‐risk human papillomaviruses (HPV) are the main etiological agents of cervical cancer. The viral early promoter regulates the expression of E6/E7 oncoproteins, and its transcriptional activity is positively or negatively regulated by host transcription factors (TFs) that are able to bind to the viral long control region (LCR). In this study, we assessed the impact of a TF library on the early transcriptional activity of high‐risk HPV‐16 and −18, and among these TF, we selected and investigated the impact of GATA3, as well as its potential role as a prognostic biomarker for the development of cervical cancer. Luciferase reporter assays demonstrated that GATA3 negatively influences the transcriptional activity of HPV‐16 and −18, downregulating E6 and E7 mRNA levels, with in silico and in vivo assays indicating that this effect is due to GATA3 direct binding to the viral LCR. Subsequently, we evaluated GATA3 levels in normal and HPV‐immortalized epithelial raft cultures and cervical cancer samples by immunohistochemistry, also accessing the TF presence in pre‐neoplastic intraepithelial cervical lesions (CIN) by immunofluorescence assays, further correlating GATA3 protein expression with the presence of HPV E6/E7 mRNA. This approach revealed an apparent inverse correlation between GATA3 expression and the grade of CIN lesions, as well as with the presence of viral oncogene transcripts. When accessing GATA3 expression in cancer samples, we observed a significant correlation between the absence of GATA3 and higher stages of cancer. Thus, our data indicates that the loss of GATA3 expression contributes to high‐risk HPV‐mediated cervical carcinogenesis, with the TF possibility acting as a protective factor whose absence enables sustained viral oncogene expression and disease progression in the cervical tissue.

**Abbreviations**
CIN: cervical squamous intraepithelial lesion
ER‐α: estrogen receptor alpha
FIGO: International Federation of Gynecology and Obstetrics
FOXA1: hepatocyte nuclear factor 3‐alpha
GATA3: trans‐acting T‐cell‐specific transcription factor GATA3
HPV: human papillomavirus
LCR: long control region
PHK: primary human foreskin keratinocytes
TF: transcription factor
TMA: tissue microarray

## 1 Introduction

Human papillomaviruses (HPV) infect skin's cutaneous and mucosal epithelium, with the viral life cycle closely tied to the differentiation program of stratified epithelia [1, 2, 3, 4]. Currently, over 400 HPV types are classified into five genera [5, 6]. The high‐risk HPV types −16, −18, −31, −33, −35, −39, −45, −51, −52, −56, −58, −59, −66, and −68 are grouped within the Alpha genus and are directly linked to the development of tumors in the cervix, vulva, vagina, penis, anal canal, and oropharynx. Notably, HPV‐16 and −18 are the most commonly detected types in cervical cancer cases [7]. HPV intratype lineages and sublineages are characterized by a 1%–2% and 0.5%–1% difference in the whole genome sequence, respectively. Specific sublineages have been associated with a higher potential for oncogenic activity [4].

The high‐risk HPV genome consists of a circular, double‐stranded DNA encoding eight protein‐coding open reading frames (ORFs)—E6, E7, E1, E2, E4, E5, L1, and L2—and a regulatory region known as the long control region (LCR) [4, 8]. The LCR contains cis‐responsive elements that regulate the activity of early promoters, specifically P97 for HPV‐16 and P105 for HPV‐18. These promoters are responsible for controlling the expression of E6 and E7 oncoproteins, which are the primary viral proteins implicated in oncogenesis [9]. The regulatory elements within the LCR can function as either activators or suppressors. Both the viral E2 protein and host transcription factors (TF), such as SP1, YY1, and CTCF, have been shown to modulate viral transcription [10, 11, 12, 13, 14].

The transcriptional environment of keratinocytes varies depending on the differentiation status of these cells across different layers of the epithelium. An imbalance in the expression of specific TFs may play a crucial role in HPV‐induced carcinogenesis [8, 15, 16, 17, 18]. GATA3 is a well‐known contributor to breast tumorigenesis [19, 20]; however, its role in cervical cancer remains unclear. GATA3 is part of the zinc‐finger GATA family of TFs, which binds to 5′‐(A/T) GATA (A/G)−3′ motifs and regulates cell identity in many tissues [21, 22, 23]. In the skin, GATA3 is vital for stem cell lineage determination and maintaining skin homeostasis during embryogenesis [24].

Additionally, GATA3 plays a significant role in adult skin by influencing the expression of several differentiation markers, such as keratin 1 and 10 [25, 26]. In a small set of samples, it was observed that GATA3 is primarily downregulated in cervical cancer tissue associated with HPV compared to normal cervical squamous epithelium [27].

Recognizing the vital role of GATA3 in squamous epithelium, we delved deeper into its relationship with cervical tissue and its influence on the transcriptional activity of the early promoter of high‐risk HPV types 16 and 18. Our findings suggest that GATA3 not only acts as a critical negative regulator of the transcription of the main HPV oncoproteins, E6 and E7, but also holds promise as a valuable prognostic biomarker for the development of cervical cancer. This insight underscores the potential of GATA3 in shaping future strategies for cervical cancer prevention.

## 2 Materials and Methods

### 2.1 Cell Culture

The cell lines used in this study included SW756 (RRID: CVCL_1727, HPV‐18 positive cervical squamous cell carcinoma), HeLa (RRID: CVCL_0030, HPV‐18 positive cervical adenocarcinoma) and SiHa (RRID: CVCL_0032, HPV‐16 positive cervical squamous cell carcinoma). These cells were cultured in Minimum Eagle Medium (MEM, Gibco, Waltham, Massachusetts, USA) supplemented with 10% heat‐inactivated fetal bovine serum (Invitrogen, Waltham, Massachusetts, USA). CaSki (RRID: CVCL_1100, HPV‐16 positive metastatic cervical squamous cell carcinoma) cells were grown in Roswell Park Memorial Institute medium (RPMI, Gibco, Waltham, Massachusetts, USA) containing 10% heat‐inactivated fetal bovine serum, and C33A (RRID: CVCL_1094, HPV negative cervical squamous cell carcinoma) cells were maintained in Dulbecco's Modified Eagle Medium (DMEM, Gibco, Waltham, Massachusetts, USA), also supplemented with 10% heat‐inactivated fetal bovine serum. Primary human foreskin keratinocytes (NHEK‐Neo #00192906, lot. 335.184, Lonza, Basel, Switzerland) were transfected with the pLNSX vector containing the complete *E6* and *E7* ORFs of HPV‐16 A1, HPV‐16 D2, or of HPV‐18 A1 sublineage variants, as previously described [28, 29]. These keratinocytes were cultivated in Keratinocyte Serum Free Medium (KSFM, Gibco, Waltham, Massachusetts, USA). All cell lines were maintained at 37°C in a humidified incubator with 5% CO_2_.

All cell lines used in this study have been annually authenticated with the GenePrint 10 System (Promega, Madison, Wisconsin, USA), following the standard methodology determined by the ATCC (ASN‐0002, American Tissue Culture Collection). Briefly, total genomic DNA was extracted from SiHa, CaSki, HeLa, SW756 and NHEK‐Neo cells by organic phenol‐chloroform purification and amplified following the instructions provided by the manufacturer. The samples were analyzed by capillary electrophoresis fragment analysis with the ABI 3730 DNA Analyzer (Applied Biosystems, Waltham, Massachusetts, USA). The authentication results were compared with standard samples from public databases (DSMZ, ATCC, JCRB, and RIKEN).

Cell lines were also periodically tested for mycoplasma infection by specific PCR amplification, with all experiments performed with mycoplasma‐free cultured cells.

### 2.2 Plasmids and Cloning

Reporter pGL3‐Basic vectors (Promega, Madison, Wisconsin, USA) containing the complete sequence of the LCR up to the early promoter (P105 for HPV‐18 and P97 for HPV‐16) of HPV‐18 A1 (nucleotide positions 7137‐105, GenBank: X05015.1), HPV‐16 A1 (nucleotide positions 7157‐97, GenBank: K02718.1) or HPV‐16 D2 (nucleotide positions 7157‐97, GenBank: AY686579.1) sublineages cloned upstream the firefly luciferase gene were previously constructed [17]. Additionally, the pCMV‐XL5 vector, which contains the complete cDNA sequence of GATA3 (SC300437, GenBank: NM_001002295, Origene, Rockville, Maryland, USA), and the empty control vector without the cDNA construct (PCMV6XL5, Origene, Rockville, Maryland, USA) were obtained from a plasmid repository and sequenced to ensure accuracy. The pGL4.75 [hRLuc/CMV] expression plasmid, containing the luciferase of *Renilla reniformis* (Promega, Madison, Wisconsin, USA) was used to evaluate transfection efficiency and for reporter assay normalization.

### 2.3 Transcription Factor Motif Analysis

Putative GATA3 binding sites within the LCR of HPV‐18 A1, HPV‐16 A1 and HPV‐16 D2 sublineages were identified in silico using different databases. The analysis utilized the TF binding motif database JASPAR CORE 2021 (https://jaspar.genereg.net [30]), the MoLo Tool from HOCOMOCO v.11 (https://hocomoco11.autosome.org [31]), and the FIMO (*Find Individual Motif Occurrences*) from the MEME Suite 5.5.3 database (https://meme-suite.org/meme/tools/fimo [32]). To further validate the consensus GATA3 binding motif identified in this analysis, we aligned it with the LCR sequence of each HPV type and sublineage obtained from the *Papillomavirus Episteme* database (PaVe, https://pave.niaid.nih.gov [33]) and the NCBI Nucleotide Bank (https://www.ncbi.nlm.nih.gov).

### 2.4 Transfections

For dual luciferase assays, cells were co‐transfected using the Fugene 6 Transfection Reagent (Promega, Madison, Wisconsin, USA) in 6‐well culture plates (Corning, Steuben County, New York, USA). The following amounts of plasmid DNA were used: 1 μg of pGL3‐LCR‐luc of each HPV type and sublineage, 300 ng of pCMV‐GATA3 or the empty vector, and 30 ng of pGL4.75 in a total of 1.33 μg of DNA, and a proportion of transfection reagent: DNA of 3:1. C33A, SiHa and Caski cells were seeded at a density of 1,000,000 cells per well, while HeLa and SW756 cells were seeded at 800,000 cells per well.

For GATA3 overexpression assays, C33A, SiHa and Caski cells were seeded at a density of 1,000,000 cells per well, while HeLa and SW756 cells were seeded at 800,000 cells per well; and transfected with 900 ng of the pCMV‐GATA3 plasmid or the empty vector.

### 2.5 Dual Luciferase Reporter Assays

Cells were harvested 48 h after transfection, and a dual luciferase assay was performed using the Dual‐Glo Luciferase Assay System (Promega, Madison, Wisconsin, USA), following the manufacturer's protocol. Relative light units (RLU) were measured using the GloMax Explorer Multimode Microplate Reader (Promega, Madison, Wisconsin, USA). Renilla luciferase measurements served as an internal control for normalizing transfection efficiency. The fold change was calculated as follows: within each independent experiment, firefly luciferase RLU values were first normalized to the corresponding Renilla luciferase RLU to correct for transfection efficiency. The resulting ratio for each GATA3‐overexpressing condition was then divided by that of the matched empty expression vector (pCMV‐XL5, OriGene, without transcription factor cDNA insert), establishing a fold‐change baseline of 1.0. This per‐experiment normalization was applied prior to averaging across biological replicates, as absolute luciferase values show inherent variability between independent transfection experiments. These experiments were conducted in triplicate, with a total of four individual experiments performed for each HPV type.

### 2.6 Quantitative Reverse Transcription Polymerase Chain Reaction (qRT‐PCR)

Total mRNA was isolated from SW756, HeLa, SiHa, and CaSki cells, both transient transfected with the pCMV‐GATA3 or the empty vector and non‐transfected. The cells were harvested 48 h after transfection. The total RNA was extracted by TRIzol (Guanidinium thiocyanate‐phenol‐chloroform extraction, Life Technologies, Waltham, Massachusetts, USA) according to the manufacturer's protocol. Additionally, total mRNA from PHK, PHK HPV‐16 A1 E6/E7, PHK HPV‐16 D2 E6/E7, and PHK HPV‐18 A1 E6/E7 cells were extracted using this same method. The quantity and quality of the mRNA were assessed using Nanodrop One (Thermo Fisher, Waltham, Massachusetts, USA). Assays were conducted with the GoTaq 1‐Step RT‐qPCR System (Promega, Madison, Wisconsin, USA) and the 7500 Real Time PCR System (Applied Biosystems, Waltham, Massachusetts, USA), following the manufacturer's instructions. The oligonucleotides used for the amplification of GATA3, GAPDH, and the E6 and E7 genes of HPV‐16 and HPV‐18 are described in Supplementary Table 1.

### 2.7 Chromatin Immunoprecipitation Assays (ChIP)

Chromatin immunoprecipitation (ChIP) assays were conducted using SW756 and SiHa cells that were transfected to overexpress GATA3. Additionally, C33A cells were co‐transfected with the pCMV‐GATA3 and pGL3‐LCR‐16 D2 plasmids. The assays were performed using the Magnify Chromatin System (Cat# 492024, Invitrogen, Waltham, Massachusetts, USA) following the manufacturer's protocol. For chromatin preparation, 3,000,000 cells from each cell line were washed with 1× PBS, treated with Trypsin/EDTA 1× (Gibco, Waltham, Massachusetts, USA), and then centrifuged at 1500 rpm for 5 min. The cell pellets were washed twice with 1X PBS, fixed with 1% formaldehyde, and subsequently quenched with 1.25 M glycine. Next, cell lysis was achieved using the Lysis Buffer included in the ChIP kit, supplemented with 1× protease inhibitors (Invitrogen, Waltham, Massachusetts, USA). Chromatin was then fragmented using the Fisherbrand Model 50 Sonic Dismembrator (Fisher Scientific, Waltham, Massachusetts, USA) with six cycles of 15 s each at 40% amplitude, with a 1‐min pause between cycles. The size of the fragmented chromatin (100–200 bp) was confirmed by electrophoresis on an Ultra‐Pure 2% agarose gel (Invitrogen, Waltham, Massachusetts, USA).

Immunoprecipitations were performed using 2 µg of rabbit anti‐GATA3 (ab199428, Abcam, Cambridge, UK) and rabbit anti‐IgG (Invitrogen, Waltham, Massachusetts, USA) as negative control. The oligonucleotides used for amplifying the 5′ and 3′ segments of the HPV‐18 A1 LCR, as well as the 5′ and central segments of the HPV‐16 A1 and D2 sublineages LCR, are detailed in Table S2. RT‐qPCR assays were conducted using the GoTaq qPCR System (Promega, Madison, Wisconsin, USA), following the manufacturer's instructions. The binding enrichment was determined by the binding over Input percentage (% Input = 2(‐ΔCq [normalized ChIP])). The normalized ChIP values correspond to the corrected input adjusted by the dilution factor. The data represented are the mean and SD from three independent experiments. Statistical significance was determined using Two‐way ANOVA, followed by Bonferroni's post‐test (*p* < 0.01 or *p* < 0.05), comparing the GATA3 group to the IgG group of each LCR region. A total of three independent assays were performed to assess the percentage of GATA3 binding.

### 2.8 Organotypic Raft Culture

Raft cultures derived from primary human keratinocytes (PHK) transducing or not transducing HPV‐16 or HPV‐18 E6/E7 were established as previously described [29, 34]. In summary, the cells were seeded on a layer of irradiated mouse fibroblasts mixed with rat tail collagen type I (BD Biosciences, Franklin Lakes, New Jersey, USA). The rafts were maintained at a liquid‐air interface, and a high‐nutrient culture medium supplemented with serum and calcium for 12 days. The resulting organotypic raft cultures were fixed with 2% formaldehyde, embedded in paraffin, and then sent for histological trimming at the Department of Pathology at ICESP (São Paulo, São Paulo, Brazil).

### 2.9 Clinical Tissue Samples

The cohort was initially established to include women diagnosed with cervical cancer who were referred to the Instituto do Cancer do Estado de São Paulo between 2008 and 2012. Follow‐up continued until December 2015. The average age of the women included in the cohort was 50.8 years, with 71% of the patients being white, 58.5% single, and 40.6% married or in a civil partnership [35]. A total of 245 samples with available formalin‐fixed paraffin‐embedded (FFPE) tissue were included in four tissue microarrays (TMA) and were histologically classified based on the FIGO 2018 staging system [36]. Ethical approval for the study was obtained from the Ethical Committee of the Medical School of the University of São Paulo (CEP n° 34718). Tissue samples derived from cervical intraepithelial neoplasia (CIN) were collected from the Hospital de Clinic in Barcelona, Spain (BCN project ID Reg HCB/2018/0210; 37) and samples from an archival collection maintained at the Department of Pathology of the University of Cambridge [37, 38]. Ethical approval for the use of these clinical samples was obtained from the Institutional Review Board of both institutions following the Human Tissue Act [39]. The samples were fixed with 10% neutral buffered formalin (NBF), dehydrated using a gradient of ethanol concentrations, paraffin‐embedded, and subsequently sectioned onto super frost poly‐l‐lysine coated slides (Gibco, Waltham, Massachusetts, USA). Hematoxylin‐eosin (H&E) staining procedures were performed on the first and last slides of each section.

### 2.10 Immunohistochemistry (IHQ) and Immunofluorescence

The immunohistochemistry signal detection was conducted using the Novolink Polymer Detection Systems kit (Leica, Wetzlar, Germany). The primary antibody employed was the rabbit anti‐GATA3 (Anti‐GATA3 antibody [EPR16651] ‐ ChIP Grade, ab199428, Abcam, Cambridge, UK) at a dilution of 1:200. Immunofluorescence assays were conducted following established protocols [37, 40]. In summary, FFPE tissue sections were first deparaffinized with 100% fresh xylene and then rehydrated using ethanol. The sections were incubated in a 1× antigen retrieval solution (eBioscience™ IHC Antigen Retrieval Solution High pH 10×, cat# 00‐4956‐58, Invitrogen, Waltham, Massachusetts, USA) for 10 min at room temperature, followed by heat‐induced epitope exposure using Biocare Medical techniques. Next, the sections were blocked with a 2.5% normal horse serum (NHS) solution at room temperature for 20 min.

For specific GATA3 staining, the primary antibody, rabbit anti‐GATA3 (Anti‐GATA3 antibody [EPR16651] ‐ ChIP Grade, ab199428, Abcam, Cambridge, UK), was diluted 1:100 and applied to each sample at room temperature for 1 h in a humidity chamber. The DAPI counterstain was also included at a dilution of 1:200. Following this, the secondary conjugated antibody (Alexa Fluor 594 goat anti‐rabbit IgG, A11012, Invitrogen, Waltham, Massachusetts, USA) was prepared at a dilution of 1:150, and the slides were incubated again at room temperature for 1 h in a humidity chamber. Finally, the slides were washed as described in previous protocols and mounted in Citofluor AF1 (cat# 17970‐25, Citofluor Ltd., Losser, Holland) using a 1.5 mm coverslip.

### 2.11 RNA In Situ Hybridization

The RNA‐ISH RNAscope (Advanced Cell Diagnostics, Newark, California, USA) methodology was employed to detect viral transcripts of high‐risk HPV in FFPE tissue sections, following the manufacturer's protocol. Initially, the FFPE slides were completely deparaffinized. Each sample was then incubated with RNAscope Hydrogen Peroxide for 10 min at room temperature, followed by antigen retrieval at 110°C for 15 min using 1× RNAscope Target Retrieval Solution. During the probe hybridization step, the samples were treated with RNAscope Protease Plus reagent in a preheated hybridization oven (HybEZ Oven, cat# 310010, Advanced Cell Diagnostics, Newark, California, USA) set to 40°C. The slides were subsequently incubated with the RNAscope Probe‐HPV‐HR18 (cat# 312591, Advanced Cell Diagnostics, Newark, California, USA) or custom probes for the detection of specific high‐risk types (HPV‐16 cat# 311521, HPV‐18 cat# 311531, HPV‐31 cat# 311551, Advanced Cell Diagnostics, Newark, California, USA), followed by a signal amplification process. Finally, for the detection of the fluorescent signal, slides were incubated for 30 min at 40°C with the Multiplex TSA Buffer provided in the kit diluted at 1:1500 with the Fluorescence Amplification Reagent (Perkin Elmer, Shelton, Connecticut, USA) and the DAPI counterstain.

### 2.12 Sample Imaging

The fluorescent sample slides were scanned using the Pannoramic MIDI Slide Scanner (3DHistech Ltd, Budapest, Hungary). The scanner was configured for fluorescent imaging with a 20× ZEISS objective (Carl Zeiss, Oberkochen, Germany) and utilized the following channels: DAPI, FITC and Rhodamine. For imaging H&E stains, the scanner was set to the brightfield mode, using the default scanning settings. The tissue microarray samples were scanned with the Axioscan 7 (Carl Zeiss, Oberkochen, Germany), set for bright‐field imaging with 20x and 10x ZEISS objectives.

### 2.13 Statistical Analysis

All statistical analyses were performed using PRISM GraphPad 9 (Analytical Software, Boston, Massachusetts, USA). The data are presented as mean and standard deviation (SD). To compare the results between the control and experimental groups, a one‐way ANOVA variance test with Bonferroni's post‐test was utilized. Overall survival (OS) was performed by Kaplan‐Meier estimation and compared with a Chi‐squared test. Clinical data were associated with Fisher's exact or Chi‐squared test, with a significance level of 5%.

## 3 Results

### 3.1 Transcription Factor Array

To identify new regulators of HPV‐16 and HPV‐18 transcription, we conducted a screening assay to assess the impact of 564 human transcription factors (TFs) on the early promoter activity of HPV‐16 (P97) and HPV‐18 (P105). The HPV‐16 A1 and D2 sublineages and the HPV‐18 A1 sub lineage were selected because they represent the molecular variants most frequently associated with differential risks of persistent infection and progression to high‐grade cervical lesions. Prior work from our group also demonstrated that transcription factors can exert differential effects on the early promoter activity of distinct HPV‐16 sublineages [17, 41]. With that, we identified 130 TFs that exhibited differential effects on the transcriptional activity of both viral types, of which 70 TFs significantly altered the basal activity of the respective HPV‐16 or HPV‐18 long control region (LCR) by two‐fold or more (Figure S1). Among these TFs, we selected GATA3 for further investigation, as previous studies have indicated that it acts as a negative regulator of HPV‐18 transcription [17]. Interestingly, the downregulation of GATA3 has been associated with an increase in lesion grade in cervical squamous intraepithelial lesions (CIN) linked to high‐risk HPV [27], while GATA3 expression is found in the normal squamous epithelium as a possible regulator of skin differentiation [24, 42].

### 3.2 GATA3 Downregulates the Transcriptional Activity of the Early Promoters of Both HPV‐16 and HPV‐18

**Figure 1:**
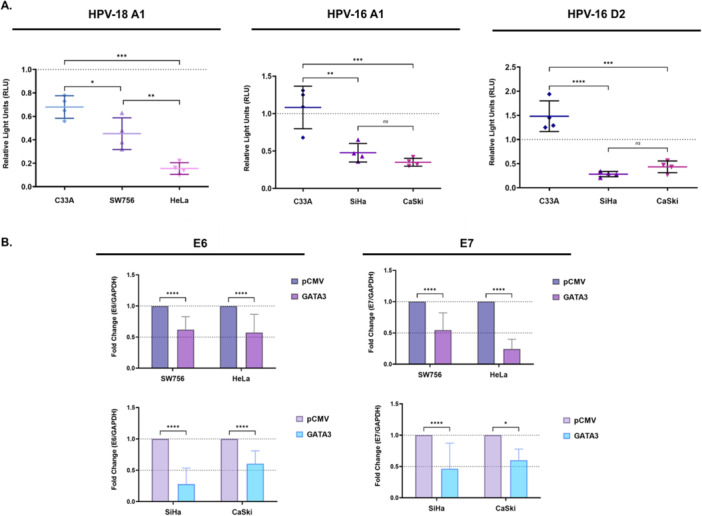
Impact of GATA3 overexpression on the transcriptional activity of the early promoter of HPV‐18 A1, HPV‐16 A1, and HPV‐16 D2 sublineages. (A) Dual luciferase promoter activity assays evaluating the effect of GATA3 overexpression on the transcriptional activity of the HPV early promoter for the HPV‐18 A1, HPV‐16 A1, and HPV‐16 D2 sublineages, performed in C33A (HPV‐negative), SiHa and CaSki (HPV‐16 positive), and SW756 and HeLa (HPV‐18 positive) cell lines. Cells were co‐transfected with the pGL3‐LCR‐luciferase reporter construct corresponding to each HPV sublineage together with either the pCMV‐GATA3 expression vector or the pCMV‐XL5 empty vector (without GATA3 cDNA insert) as negative control. Transfection efficiency was normalized in each experiment using *Renilla* luciferase activity (pGL4.75 [hRLuc/CMV]). Data are expressed as fold‐change relative to the normalized luciferase activity (Firefly/*Renilla* ratio) of cells co‐transfected with the empty expression vector, which is set to 1.0 (dashed line). Fold‐change values were calculated within each independent experiment prior to averaging across replicates. Each bar represents the mean ± SD of four independent experiments, each conducted in triplicate. Both the empty vector control (value = 1.0) and the GATA3‐overexpressing condition are shown for each cell line and sublineage. Statistical analysis was performed using one‐way ANOVA with Bonferroni's post‐test; ****p < 0.0001. (B) Quantitative real‐time RT‐PCR measuring the mRNA expression levels of the HPV oncogenes *E6* (left panels) and *E7* (right panels) in HPV‐16 positive cells (SiHa and CaSki; upper panels) and HPV‐18 positive cells (SW756 and HeLa; lower panels) following transient overexpression of GATA3, compared to cells transfected with the empty expression vector (mock control). Expression levels of *E6* and *E7* were normalized to the housekeeping gene *GAPDH* and calculated using the 2^−ΔΔCq^ method. Each bar represents the mean ± SD of three independent experiments. Statistical analysis was performed using two‐way ANOVA with Bonferroni's post‐test; ***p < 0.001.

To validate the GATA3 data obtained from the initial screening assay (Figure S1C), we assessed the impact of GATA3 on the transcriptional activity of the HPV‐16 and HPV‐18 early promoters by ectopically overexpressing GATA3 in the HPV‐negative C33A cell line and in the HPV‐18 positive cell lines SW756 and HeLa, as well as in the HPV‐16 positive cell lines SiHa and CaSki. Each cell line was co‐transfected with the corresponding pGL3‐LCR‐luciferase reporter construct together with either the pCMV‐GATA3 expression vector or the empty pCMV‐XL5 vector (without GATA3 cDNA insert), which served as the negative control (value = 1.0; Figure 1A). Data are expressed as fold‐change relative to the normalized luciferase activity of the empty vector condition, calculated within each independent experiment after normalization to *Renilla* luciferase to correct transfection efficiency. GATA3 overexpression led to a decrease in HPV‐18 P105 early promoter activity in all cell lines tested (Figure 1A). Transcriptional repression was markedly more pronounced in the HPV‐18 positive cell lines, with approximately 50% reduction in SW756 cells and approximately 90% reduction in HeLa cells (*p* < 0.0001), compared to a modest reduction observed in the HPV‐negative C33A cells. Regarding the HPV‐16 P97 promoter, GATA3 overexpression produced significant repression in both SiHa and CaSki cells for the A1 and D2 sublineages (*p* < 0.0001). In C33A cells transfected with the HPV‐16 A1 or D2 LCR–reporter construct, a slight increase of approximately 1.3‐fold in P97 activity was observed for both sublineages, in contrast to the repression observed in HPV‐16 positive cells.

### 3.3 GATA3 Suppresses the Expression of HPV E6 and E7

**Figure 2:**
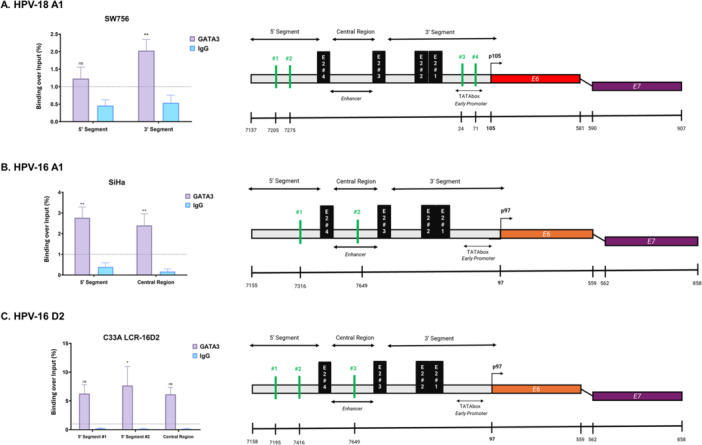
Chromatin Immunoprecipitation assays evaluating in vivo binding of GATA3 within the LCR of HPV‐18 A1, HPV‐16 A1 and HPV‐16 D2 sublineages. (A) GATA3 binding enrichment in the 5′ and 3′ segments of the HPV‐18 A1 LCR, indicating significant binding motifs located in the distal segment which is close to the early promoter. This is illustrated in the accompanying schematic figure of the LCR beside the graph. The green numbered bars indicate each GATA3 binding site, as predicted by in silico analysis. (B) Enrichment of GATA3 binding to 5′ and central segments of the HPV‐16 A1 LCR. Both segments demonstrated significant enrichment following immunoprecipitation. (C) Enrichment of GATA3 in the 5′ segment and central segments of the HPV‐16 D2 LCR. Significant binding of GATA3 was solely observed for the 5′ segment #2. Binding enrichment was determined by the binding over input percentual (% Input = 2(‐ΔCq [normalized ChIP])). The normalized ChIP values correspond to the corrected input Cq value adjusted by the twofold dilution factor. The data represented are the mean and SD from three independent experiments. Statistical significance was determined using Two‐way ANOVA, followed by Bonferroni's post‐test (*p* < 0.01 or *p* < 0.05), comparing the GATA3 group to the IgG group of each LCR region.

Next, we analyzed the levels of *E6* and *E7* mRNA, which are directly regulated by the early promoter, in the HPV‐16 positive (SiHa and CaSki) and HPV‐18 positive (HeLa and SW756) cells. As expected, when we compared the *E6* and *E7* mRNA levels in cells transfected with the pCMV‐empty vector to those transfected with pCMV‐GATA3, we observed a significant reduction in *E6* and *E7* mRNA levels in all cell lines that overexpressed GATA3 (Figure 1B). The overexpression of GATA3 in these cell lines was confirmed by qPCR (Figure 2). This finding further supported the negative effect of GATA3 on HPV‐16 and HPV‐18 transcription that was previously observed in the reporter assays.

### 3.4 GATA3 Directly Binds to the LCR of HPV‐16 and −18

Host TFs that bind to specific motifs located within the viral LCR are more likely to either activate or impair the activity of the HPV early promoters. To verify that the effect of GATA3 was due to the direct binding of the TF to the viral long control region, we scanned the complete LCR sequences of the HPV‐16 A1, HPV‐16 D2, and HPV‐18 A1 sublineages to identify putative GATA3 binding sites. This analysis led to the identification of the motifs detailed in Table 1. Additionally, we examined the LCR of other viral types and observed that the identified potential GATA3 binding sites are shared among high‐risk HPV types (Table S3 and Figure S3), suggesting that GATA3 transcriptional regulation may be conserved across high‐risk HPVs.

**Table 1:** GATA3 motifs predicted by in silico analysis of the complete long control region (LCR) sequences for the HPV‐16 A1, HPV‐16 D2 and HPV‐18 A1 sublineages. Nucleotide positions, measured in base pairs, are referenced according to the LCR sequence of each HPV type and sublineage. The JASPAR score is set to be equal or higher than 80%. FIMO *p*‐values are noted as less than 0.0001.

HPV Type	LCR Region	GATA3 Motif Sequence (5'–3')	Nucleotide Position (pb)	JASPAR Score	FIMO *p* value
HPV‐18 A1	5′ Segment	TGATTG	7205–7210	0.95	3.06E‐05
		TTAAATAAAA	7275–7285	0.85	4.78E‐05
	3′ Segment	TATATAAAAAA	24–34	0.85	6.99E‐05
		TATATAAAAG	71–80	0.85	5.76E‐05
HPV‐16 A1	5′ Segment	ATAAATAAACT	7316–7326	0.85	9.66E‐06
	Central region	TCATATAAAAT	7649–7659	0.82	6.24E‐05
HPV‐16 D2	5′ Segment	TTATATGTTTGT	7195–7206	0.85	2.85E‐05
		ACTATATTTTG	7416–7426	0.85	3.17E‐05
	Central Region	TCATATAAAAT	7649–7659	0.82	4.32E‐05

To further validate these binding sites, we conducted chromatin immunoprecipitation assays to assess the in vivo binding of GATA3 to the identified sites within the LCR. The ChIP assays were performed at SW756 cells for HPV‐18 A1 LCR binding evaluation, and SiHa cells for HPV‐16 A1, since these cell lines have integrated HPV genomes of these sublineages. For HPV‐16 D2, the experiments were performed at C33A cells transfected with the HPV‐16 D2 LCR.

For the HPV‐18 A1 sublineage, we detected GATA3 binding to the 3′ segment of the LCR, likely at putative binding sites #3 and/or #4, with a binding enrichment of twofold compared to the input control (Figure 2A).

However, no significant binding was observed at predicted binding sites within the 5′ segment. In the case of the HPV‐16 A1 sublineage, both the 5′ and central segments showed significant enrichment after precipitation, with increases of 2.7‐fold and 2.4‐fold compared to the input, respectively (Figure 2B) For the HPV‐16 D2 sublineage, GATA3 significantly bound only to the 5′ segment #2, with a sevenfold increase (*p* < 0.005) (Figure 2C).

### 3.5 GATA3 Is Downregulated in the Presence of E6/E7 Oncoproteins

**Figure 3:**
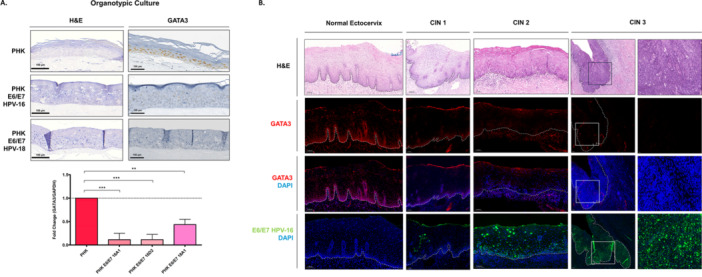
GATA3 expression is downregulated in HPV E6/E7‐transduced keratinocytes and in cervical intraepithelial lesions, and its absence correlates spatially with the presence of HPV E6/E7 mRNA. (A) Immunohistochemistry assays comparing GATA3 protein expression in organotypic raft cultures derived from uninfected primary human keratinocytes (PHK, left column) and from PHK transduced with the E6 and E7 oncoproteins of HPV‐16 A1 (center column) or HPV‐18 A1 (right column) at passage 30 (p30), at which point cells are considered immortalized. Scale bar: 100 µM. GATA3 signal was detected using a rabbit anti‐GATA3 antibody (ab199428, Abcam; 1:200). Haematoxylin was used as a nuclear counterstain. Whereas uninfected PHK raft cultures display robust basal and mid‐layer GATA3 nuclear staining, cultures expressing E6/E7 of either HPV‐16 or HPV‐18 show marked loss of GATA3 protein. The lower panel shows a RT‐qPCR analysis of GATA3 transcript levels demonstrated a decrease in GATA3 mRNA in the presence of E6/E7. The data was normalized to the GAPDH housekeeping gene and quantified using the 2^−ΔΔCt^ method, presented as mean ± SD. (B) Immunofluorescence assays performed on formalin‐fixed paraffin‐embedded (FFPE) tissue comparing the pattern of GATA3 protein expression (ab199428, 1:100) across normal ectocervical squamous epithelium (top row) and increasing grades of high‐risk HPV‐associated cervical intraepithelial neoplasia (CIN1, CIN2, and CIN3, subsequent rows). DAPI (blue) was used as a nuclear counterstain. The presence of HPV E6/E7 mRNA was assessed simultaneously by RNA in situ hybridization using the RNAscope® technology (HPV‐16 cat# 311521, HPV‐18 cat# 311531; Advanced Cell Diagnostics), as shown in the right column. Boxed areas indicate representative high‐magnification regions selected to illustrate the cellular compartment of interest; the distinct positioning of boxes between the GATA3 and RNAscope panels reflects the spatial distribution of each marker within the same tissue with different subsequent sections. Normal ectocervical epithelium (first row) serves as the baseline reference for GATA3 expression pattern.

Host TFs may play a role in the deregulation of viral transcription during cellular immortalization and transformation. As a result, abnormal expression of these proteins could significantly impact HPV‐associated carcinogenesis. To explore this, we initially aimed to compare GATA3 levels and distribution in raft cultures derived from PHK transducing or not HPV‐16 and HPV‐18 oncoproteins (Figure 3A). We observed that the pattern of GATA3 expression in uninfected keratinocytes was similar to that observed in normal ectocervical epithelium, with the protein signal most pronounced in the basal and parabasal cell layers, extending into the mid‐spinous layer, and absent in the upper, more differentiated cell layers. (Figure 3B). Here, the presence of GATA3 was most pronounced in the basal layer of the stratified tissue, extending to the middle layers, but was absent in the upper, more differentiated cells. This pattern of GATA3 expression was consistent with previous observations in the stratified epithelium of exposed skin (Masse et al., 2014).

In contrast, raft cultures derived from high‐passage (p30) PHK transduced with E6/E7 of HPV‐16 A1 or HPV‐18 A1, which are considered immortalized [17, 29], showed a loss of GATA3 expression (Figure 3A). This reduction was also evident in the mRNA levels of GATA3 in these cells. Specifically, PHK transduced with HPV‐16 E6/E7 exhibited more than a 70% decrease in GATA3 mRNA levels, while cells transduced with HPV‐18 E6/E7 showed a 50% decrease (Figure 3B). When comparing the levels of GATA3 observed in the normal PHK with the HPV+ cancer cell lines, we also observed the reduction of the TF expression in the tumoral cells (Figure S4), with the notable exception of CaSki cells, which retained detectable endogenous GATA3 expression. While CaSki cells harbor an exceptionally high HPV‐16 copy number (estimated at approximately 500–600 integrated copies per cell) [43, 44], previous studies have demonstrated that E6/E7 transcript levels in CaSki and SiHa cells are comparable [45], and that viral oncogene expression in CaSki arises from a single transcriptionally active integration event [46], rather than from the total integrated genome load. Therefore, excess oncoprotein production is unlikely to account for the coexistence of detectable GATA3 and robust E6/E7 expression in this cell line. We propose instead that the GATA3 detected in CaSki reflects its intestinal metastatic origin [47], a tissue in which GATA3 is physiologically higher [48], but where the endogenous levels fall below the functional threshold required to effectively repress viral transcription, or where the endogenous GATA3 is somehow impaired by other molecular mechanisms. This interpretation is supported by our observation that exogenous GATA3 overexpression in CaSki cells, which substantially exceeds endogenous levels, achieves significant and comparable repression of HPV‐16 P97 promoter activity to that seen in SiHa cells (Figure 1A).

### 3.6 GATA3 Levels Decrease as the Grading of High‐Risk HPV‐Induced Cervical Intraepithelial Lesions (CIN) Increases and Are Associated With FIGO Staging

We observed that the transcription activity of HPV‐16 A1, HPV‐16 D2, and HPV‐18 A1 is significantly downregulated when GATA is overexpressed in HPV‐positive cells. Additionally, we found that GATA3 is mostly absent in raft cultures derived from primary human keratinocytes (PHKs) immortalized by these HPV types and sublineages, the downregulation taking effect in both protein and mRNA detection (Figure 3A). Based on these findings, we hypothesized that the loss of this TF could play a significant role in cervical carcinogenesis induced by high‐risk HPV. Therefore, we evaluated the levels and distribution of GATA3 in various grades of cervical intraepithelial neoplasia (CIN1 to CIN3) as well as in cervical cancer samples.

Figure 3B presents an immunofluorescence assay designed to analyze GATA3 levels at CIN samples from an archival cohort maintained at the Department of Pathology of the University of Cambridge, with the simultaneous identification of normal epithelial regions alongside high‐risk HPV associated CIN of varying grades. It is notable that as the grade of the squamous cervical lesion increases, there is less expression of GATA3, with most of the CIN2 and CIN3 lesions bearing virtually no signal detection of the TF. Interestingly, this decrease in GATA3 expression in high‐grade lesion sites corresponded with an increase in the detected levels of E6/E7 mRNA (Figure 3B, last row panels), which further corroborates that the presence of GATA3 negatively impacts the expression of the viral products.

This pattern was also noted in additional samples from the same cohort when assessing specific high‐risk HPV mRNA signal (Figure S5). These findings suggest that the loss of GATA3 expression is associated with increasing cervical lesion grade and a corresponding increase in detectable E6/E7 mRNA levels. Furthermore, regions with absent or markedly reduced GATA3 signal were associated with detectable HPV E6/E7 mRNA, particularly at the basal cell layers of the epithelium, consistent with the notion that loss of GATA3 may enable sustained viral oncogene expression as the disease progresses.

**Figure 4:**
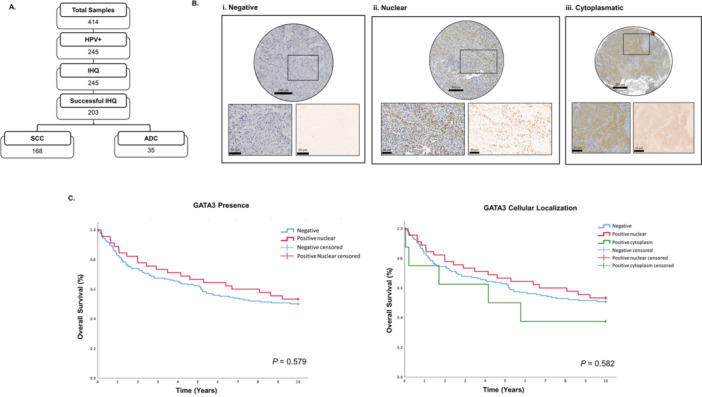
Correlation of GATA3 cellular localization with overall survival in clinical cervical cancer samples. (A) This section provides a brief overview of the samples analyzed based on a previous study, where samples were collected, characterized, and organized into tissue microarrays (TMA) (Nogueira Genta et al., 2017). (B) Characterization of GATA3 immunostaining in the TMA samples revealed that most were negative (71.4%) (panel i). Among the positive samples (28.7%), the cellular localization of the transcription factor was either nuclear (panel ii) or cytoplasmic (panel iii). Scale bar = 200 µm. Magnification panel scale = 50 µm. (C) Kaplan‐Meier of the overall survival for negative and positive GATA3 samples is presented in the left panel. Additionally, among the positive samples, survival analysis based on the status of GATA3 cellular localization is shown in the right panel.

Finally, we evaluated GATA3 levels in 245 cervical cancer TMA samples obtained from women treated at the Institute of Cancer of Sao Paulo, Brazil [31] (Figure 4A). GATA3 staining was classified into negative and positive, with positive samples further classified by the location of immunohistochemical staining as nuclear and cytoplasmic. (Figure 4B). Our findings revealed that 71.4% (120 out of 168) of the samples were GATA3 negative, while 28.7% (48 out of 168) were positive. Among the positive samples, 91.6% (44 out of 48) showed nuclear staining, whereas 8.3% (4 out of 48) exhibited cytoplasmic GATA3 localization. Among the samples analyzed, GATA3 levels and localization were not linked to any of the socio‐clinical‐pathological variables, such as age, tumor extension, or HPV type. However, we observed that while these results did not reach statistical significance (*p* = 0.579), reflecting the limited sample size, GATA3‐positive cases showed a trend towards better overall survival. This observation should be regarded as exploratory and hypothesis‐generating, requiring confirmation in larger independent cohorts. Furthermore, among the GATA3‐positive samples, those exhibiting nuclear localization of GATA3 had non‐significant (*p* = 0.582) higher overall survival than those with cytoplasmic localization (Figure 4C).

Furthermore, when examining the relationship between the localization of GATA3 and the FIGO staging of the samples (Table 2), we found that most samples with nuclear GATA3 were classified as stage IIIC or lower (29.2%, 26 out of 46 samples), and there were no samples with nuclear GATA3 localization at stage IVB. In contrast, a significant majority of samples that tested negative for GATA3 were at stage IIIC (78.9%, 60 out of 149 samples), while those with cytoplasmic GATA3 localization were predominantly at stage IVB (37.5%, 3 out of 8 samples) (*p* < 0.005).

**Table 2:** Correlation between the FIGO 2018 classification of cervical tumors and the cellular localization of GATA3 staining (*p* < 0.005). The FIGO stages were grouped according to pathological groups.

FIGO* Classification	GATA3 Localization					
	Negative		Nucleus		Cytoplasm	
	n°	%	n°	%	n°	%
IA1‐IB2	22	73.3	6	20	2	6.7
IB3, IIA, IIB, IIIA, IIIB, IVA	62	69.7	26	29.2	1	1.1
IIIC1, IIIC2	60	78.9	14	18.4	2	2.6
IVB	5	62.5	0	0	3	37.5
Total	149	73.4	46	22.7	8	3.9

*Bhatla et al., 2018.

## 4 Discussion

The relationship between host cell TFs and the regulation of HPV early promoter activity is crucial for synchronizing the viral life cycle with the differentiation program of the stratified squamous epithelium. This interaction directly influences the expression of the main viral oncoproteins, E6 and E7. Several TFs have been identified as regulators of high‐risk HPV transcriptional activity [49, 50, 51]. GATA3 serves as a pioneer regulator in the development, differentiation, and cell fate decisions in both germinal and adult tissues [52]. In the stratified squamous epithelium of the skin, GATA3 plays a crucial role in the differentiation of keratinocytes by modulating the expression of important markers involved in this process, such as keratins 1 and 10, involucrin, and loricrin [26, 42, 53].

Previously, we used a high‐throughput transfection array to assess the effects of 704 TFs upon the transcription of HPV‐16 and HPV‐18. Among other novel TFs identified that impact early viral expression, we found GATA3 to be a regulator of the transcriptional activity of HPV‐18 P105 promoter in HPV‐negative C33A cells [17]. In this study, we expanded our analysis by reevaluating the effects of approximately 500 TFs using a more precise dual luciferase transfection assay. Additionally, we conducted validation assays in cells that are positive for HPV‐16 (CaSki and SiHa) and HPV‐18 (SW756 and HeLa), where the ectopic expression of GATA3 confirmed the negative effect of the TF upon the transcriptional activity of the early promoter of both high‐risk HPV‐16 and −18, also concomitantly impacting the levels of E6 and E7 mRNA (Figure 1). Importantly, this effect was more significant in all evaluated HPV‐positive cell lines compared to the HPV‐negative C33A cells, which could be explained by the different transcriptional environment found in HPV‐positive cells. The long‐term presence of E6 and E7 oncoproteins extensively reprograms the host transcriptional landscape, reshaping the balance and activity of LCR‐binding factors such as Sp1, Sp3, and YY1 (8; 15; 16). In contrast, C33A cells maintain a distinct transcription factor repertoire, including higher relative expression of activating factors such as NF1 [16], may render the exogenous episomal LCR reporter more susceptible to transactivation than to GATA3‐mediated repression in this cellular context.

Importantly, the GATA3‐mediated repression observed in HPV‐positive versus HPV‐negative cells is consistent with a model in which GATA3 acts as a restriction factor whose repressive function is most effective in the transcriptional context of HPV‐infected cells.

Notably, the retention of endogenous GATA3 in CaSki cells in contrast to its loss in the other HPV‐positive cell lines examined could illustrate an important dose‐dependent dimension of GATA3‐mediated restriction. Although GATA3 protein is detectable in CaSki, its endogenous levels appear to fall below the functional threshold required for effective repression of the viral LCR, likely because this cell line was originally established from a cervical metastasis at the small intestine, a tissue in which GATA3 expression is physiologically elevated independently of HPV infection, and it's function could be impaired by other molecular mechanisms present in this cell lineage. The critical evidence supporting this interpretation is our finding that exogenous overexpression of GATA3 in CaSki cells, which substantially raises intracellular GATA3 above the endogenous level, achieves significant and comparable repression of HPV‐16 P97 promoter activity to that observed in SiHa cells (Figure 1A). This demonstrates that GATA3's restriction factor function is intact in CaSki once the TF is exogenously expressed and that its endogenous levels or functionally could be are sub‐threshold or impaired, reinforcing the broader model in which the progressive loss of GATA3 expression that could be driven by HPV‐associated epigenetic reprogramming in the cervical epithelium could be a key enabling event in viral oncogene deregulation and carcinogenesis. In silico binding site analysis indicated the presence of multiple distinct GATA3 binding elements within the LCR, and active binding of the TF was further confirmed through ChIP followed by qPCR assays (Figure 2).

Our analysis of GATA3 expression in organotypic keratinocyte cultures revealed that high‐risk HPV‐16 or HPV‐18 E6/E7 proteins are associated with a reduction in GATA3 expression at both protein (Figure 3A) and RNA levels (Figure 3B). The mechanism by which GATA3 is progressively silenced during HPV‐mediated carcinogenesis has not been directly elucidated. Two hypotheses may be considered. First, as observed in renal cell carcinoma, is known that GATA3 promoter hypermethylation drives its transcriptional loss [54]. Given that HPV E7 interacts with chromatin‐modifying enzymes including DNMT1 and histone deacetylases, HPV‐driven epigenetic reprogramming could secondarily silence GATA3. Second, E6/E7‐mediated disruption of p53 and pRb pathways broadly rewires keratinocyte transcription, potentially creating a cellular environment incompatible with sustained GATA3 expression. In this scenario, initial GATA3 loss would permit elevated E6/E7 expression, which in turn may further suppress GATA3, establishing a self‐reinforcing loop. These hypotheses require direct experimental investigation.

We next analyzed the expression of GATA3 in the cervical squamous epithelium and HPV‐16 intraepithelial lesions. In normal tissue, GATA3 is expressed in the basal to middle layers, consistent with previous findings in exposed skin stratified epithelium [26]. However, in HPV infected tissue, GATA3 expression appears to be downregulated as the lesion grade increases from CIN1 to CIN3 (Figure 3B), becoming largely absent in established squamous carcinomas (Figure 4B). These findings support the data reported by Steenbergen and colleagues (2002) [27], which indicated that GATA3 might play a role in the immortalization of keratinocytes mediated by the presence of HPV‐16 and −18 E6/E7, with its expression being downregulated during this process. In fact, the patterns of GATA3 expression observed in CIN lesions and cervical cancer samples reinforce the connection between GATA3 and cervical tumorigenesis and further indicate that the role of GATA3 might also be significant in tumorigenesis caused by other high‐risk HPVs.

We observed that CC samples with negative GATA3 staining showed a trend towards worse overall survival compared to samples with positive GATA3 staining (Figure 4C). Additionally, since transcription takes place in the nucleus, it is important to consider the cellular localization of GATA3 in tumors in which this protein is detected [55]. Among GATA3‐positive CC samples, those exhibiting cytoplasmic GATA3 staining had a worse overall survival rate compared to those with nuclear GATA3 staining. However, these observations should be interpreted with caution, as the data did not reach statistical significance. This may be partly due to the small number of CC cases evaluated in the current study, which may constitute an important limitation in the ability to detect a subtle association between GATA3 absence and survival. When analyzing the correlation between GATA3 absence and the tumor staging, the absence of GATA3 was significantly associated with more advanced FIGO grading [32] (Table 2).

The association between GATA3 and cervical tumorigenesis may involve mechanisms previously observed in other cancers where this TF plays a significant role. For instance, in breast cancer, GATA3 is linked to improved prognosis and overall survival [55, 56], especially when positively correlated with the presence of estrogen receptor alpha (ER‐α) [57, 58, 59]. GATA3 responds to estrogen and establishes a positive feedback loop with ER‐α and the transcription factor FOXA1 in mammary tissue [59]. Cervical cancer is also highly responsive to estrogen, which correlates with increased levels of E6 and E7 proteins [60]. Additionally, FOXA1 is associated with the enhanced production of oncoproteins and acts as a positive regulator of early promoter activity [17]. Investigating the GATA3/ER‐α/FOXA1 axis in the cervix is crucial for understanding the role of GATA3 in HPV‐related tumor development in this region. This research could also help in advancing GATA3 as a potential target for cervical cancer treatment.

Based on the data presented, we propose that the progressive loss of GATA3 represents a key event in HPV‐mediated cervical carcinogenesis, rather than GATA3 playing a proactive role in tumor promotion. GATA3 functions as a direct transcriptional repressor of viral E6/E7 oncogene expression; accordingly, its absence removes a critical constraint on oncoprotein production, thereby enabling cell immortalization and malignant progression. Finally, GATA3 has the potential to serve as a valuable prognostic biomarker; however, further studies involving more cases of cervical cancer are warranted.

## Author Contributions

Laura Sichero designed the study. Laura Sichero and Valéria Talpe‐Nunes drafted the original manuscript. Valéria Talpe‐Nunes conducted experiments. Valéria Talpe‐Nunes, Laura Sichero, and Aline Lopes Ribeiro contributed to the results interpretation. Valéria Talpe‐Nunes, João Simão Sobrinho, and Amanda Schiersner Caodaglio conducted plasmid amplification and library preparation. Rossana Veronica Mendonza Lopez contributed to the statistical analysis. Rafaella Almeida Nunes and Thais Rocha contributed to the histological analysis. Maria Luiza Nogueira Dias Genta contributed to the acquisition of CC clinical samples. Konstanze Schichl, Ademola Aiyenuro, and John Doorbar contributed to CIN sample acquisition and immunohistochemistry results analysis. All authors commented and revised the manuscript and approved the final manuscript.

## Ethics Statement

Ethical approval for the use of cervical cancer samples was obtained from the Ethical Committee of the Medical School of the University of São Paulo, São Paulo, Brazil (CEP n° 34718). Ethical approval for the use of clinical cervical lesion samples was obtained from the Institutional Review Board of the Hospital de Clinic, Barcelona, Spain (BCN project ID Reg HCB/2018/0210), following the Human Tissue Act at the Department of Pathology of the University of Cambridge.

## Conflicts of Interest

The authors declare no conflicts of interest.

## Supporting information

Supporting File 1

Supporting File 2

Supporting File 3

Supporting File 4

Supporting File 5

Supporting File 6

## Data Availability

All data presented at this manuscript is presented at the main figures, and supplementary information is available in the Supplementary Data section.
