# Supplementary material for: GATA3 Inhibits the Expression of Viral E6/E7 Genes, and Its Expression Is Compromised During HPV‐Mediated Cervical Carcinogenesis
Source: J Med Virol. 2026 Jun 26;98(7):e71034. doi: 10.1002/jmv.71034 (PMC13306532; doi:10.1002/jmv.71034)
Supplement: Supplementary file 1 — Supporting File 1 [file JMV-98-e71034-s003.docx]

**SUPPLEMENTARY TABLES**

**Supplementary Table 1. Oligonucleotide sequences of the primers used for RT-qPCR amplification of GATA3, the housekeeping gene GAPDH, and E6 and E7 mRNA of HPV-16 and HPV-18**. TA, annealing temperature, amplicon size in base pairs, F: forward, R: reverse.

| **Target** | **Nucleotide Position*** | **Primer Sequence (5' - 3')** | **TA (°C)** | **Amplicon (bp)** |
| --- | --- | --- | --- | --- |
| **GATA3** | 900 - 1015 | F: GCGGGCTCTATCACAAAATGA | 60 | 115 |
|  |  | R: GCCTTCGCTTGGGCTTAAT |  |  |
| **HPV-16 E6** | 124 - 300 | F: GCGACCCAGAAAGTTACCACAG | 60 | 176 |
|  |  | R: GCATAAATCCCGAAAAGCAAAG |  |  |
| **HPV-16 E7** | 682 - 802 | F: AGAACCGGACAGAGCCCATTAC | 60 | 120 |
|  |  | R: GCCCATTAACAGGTCTTCCAAAG |  |  |
| **HPV-18 E6** | 299 - 656 | F: CGATTTCACAACATAGCTGGGC | 60 | 357 |
|  |  | R: GAGTCGTTCCTGTCGTGCTC |  |  |
| **HPV-18 E7** | 885 – 621 | F: TTACTGCTGGGATGCACACC | 60 | 303 |
|  |  | R: ATGCATGGACCTAAGGCAAC |  |  |
| **GAPDH** | 942 - 1117 | F: AAGGTGGTGAAGCAGGCGT | 60 | 176 |
|  |  | R: GAGGAGTGGGTGTCGCTGTT |  |  |

*Relative to the complete nucleotide sequence of each target.

**Supplementary Table 2. Oligonucleotide sequences of the primers used for ChIP-qPCR assays to access the enrichment of GATA3 motifs within the LCR of HPV-18 A1, and HPV-16 A1 or HPV-16 D2 sublinages**. TA, annealing temperature, amplicon size in base pairs F: forward, R: reverse.

| **Target** | **Primer Sequence (5' - 3')** | **TA (°C)** | **Amplicon (bp)** |
| --- | --- | --- | --- |
| **HPV-18 A1 5' Segment** | F: GTATGTCCTGTGTTTGTGTT | 60 | 143 |
|  | R: GGCGCAACCACATAACACAC |  |  |
| **HPV-18 A1 3' Segment** | F: GACTAAGCTGTGCATACATA | 60 | 167 |
|  | R: TATTGTGGTGTGTTTCTCACA |  |  |
| **HPV-16 A1 5' Segment** | F: GTATGTATGGTATAATAAACACGTGT | 59.8 | 151 |
|  | R: AACAGGATGTAGCAAATATAGTT |  |  |
| **HPV-16 A1 Central Region** | F: CTGCACTATGTGCAACTACTGAA | 59 | 167 |
|  | R: CATTTAGTTGGCCTTAGAAGTT |  |  |
| **HPV-16 D2 5' Segment#1** | F: GTTTATATGTTTGTATGTGCTTGTAT | 60 | 168 |
|  | R: CAATTAGTAGGTGATGAAACAATA |  |  |
| **HPV-16 D2 5' Segment#2** | F: TATTGTTTCATCACCTACTAATTGTG | 61 | 141 |
|  | R: ATGCAACCGAATTCGGTTGAAG |  |  |
| **HPV-16 D2 Central Region** | F: CACTATGTGCAACTACTGAATCA | 60 | 200 |
|  | R: GAACAATGTATGACTAACCTTTACAC |  |  |

**Supplementary Table 3. Predicted GATA3 binding sites at the LCR of high-risk Alpha HPV types.** The putative binding sites were analyzed with the MoLo Tool available at the HOCOMOCO platform (p < 0.002) and further validated by the FIMO algorithm (p < 0.004).

| **HPV Type** | **GenBank Accession n°** | **GATA3 Motif Sequence (5’ – 3’)** | **Start** | **End** | **-log10(P-value)** | **P-value** |
| --- | --- | --- | --- | --- | --- | --- |
| **HPV-16** | K02718 | TATATAAAA | 247 | 255 | 2,09 | 8.128e-3 |
|  |  | CATATAAAA | 498 | 506 | 2,155 | 6.998e-3 |
| **HPV-18** | X05015 | ATGATTGCA | 67 | 75 | 2,047 | 8.974e-3 |
|  |  | GTTATAAAA | 259 | 267 | 2,012 | 9.727e-3 |
|  |  | TATATAAAA | 744 | 752 | 2,09 | 8.128e-3 |
|  |  | TATATAAAAG | 791 | 799 | 2,09 | 8.128e-3 |
| **HPV-31** | J04353 | AATATAATA | 631 | 639 | 2,103 | 7.889e-3 |
|  |  | TAGATAATC | 681 | 689 | 3,332 | 4.656e-4 |
| **HPV-33** | M1273 | AACATAACA | 15 | 23 | 2,336 | 4.613e-3 |
|  |  | AACATAACA | 101 | 109 | 2,336 | 4.613e-3 |
| **HPV-35** | X7447 | CACATAACA | 29 | 37 | 2,349 | 4.477e-3 |
|  |  | CACATAATA | 56 | 64 | 2,233 | 5.848e-3 |
|  |  | CATATAAAG | 356 | 364 | 2,151 | 7.063e-3 |
|  |  | CACATAATA | 597 | 605 | 2,233 | 5.848e-3 |
| **HPV-39** | M62849 | CATATAATA | 166 | 174 | 2,12 | 7.586e-3 |
| **HPV-45** | X74479 | TACATAAAA | 729 | 737 | 2,207 | 6.209e-3 |
| **HPV-51** | M62877 | AAGATAAAA | 605 | 613 | 3,78 | 1.660e-4 |
|  |  | GTTATAAGA | 760 | 768 | 2,181 | 6.592e-3 |
|  |  | CATATAAAA | 831 | 839 | 2,155 | 6.998e-3 |
| **HPV-52** | X74481 | TACATAACA | 77 | 85 | 2,289 | 5.140e-3 |
|  |  | TACATAACA | 117 | 125 | 2,289 | 5.140e-3 |
|  |  | AACATAAGA | 220 | 228 | 2,393 | 4.046e-3 |
|  |  | AACATAACA | 225 | 233 | 2,336 | 4.613e-3 |
| **HPV-56** | X74483 | CAGATAAAA | 555 | 563 | 3,881 | 1.315e-4 |
|  |  | CATATAAAA | 813 | 821 | 2,155 | 6.998e-3 |
| **HPV-58** | D90400 | TACATAAAA | 42 | 50 | 2,207 | 6.209e-3 |
|  |  | TATATAAAA | 673 | 681 | 2,09 | 8.128e-3 |
| **HPV-59** | X77858 | CATATAAAG | 783 | 791 | 2,151 | 7.063e-3 |
| **HPV-66** | U31794 | CAGATAAAA | 483 | 491 | 3,881 | 1.315e-4 |
|  |  | CATATAAAA | 731 | 739 | 2,155 | 6.998e-3 |
| **HPV-68** | DQ080079 | CATATAACA | 1 | 9 | 2,246 | 5.675e-3 |
|  |  | CATATAATA | 207 | 215 | 2,12 | 7.586e-3 |

**SUPPLEMENTARY FIGURES LEGENDS**

**SUPPLEMENTARY FIGURE 1. Transcription factor screening assay. (A)** Heatmap of the 130 transcription factors (TFs) identified through the dual luciferase reporter promoter activity that impacted the transcription of HPV-16 A1, HPV-16 D2 and HPV-18 A1 sublineages in C33A cells. The heatmap displays the mean of the normalized relative light units (RLU) from two independent experiments. The heatmap was generated in RStudio (v.3.3.4) with Tidy Heatmap package (Engler 2024, https://jbengler.github.io/tidyheatmaps/). The distribution of TFs identified in the screening assay to affect HPV-16 A1, HPV-16 D2 and HPV-18 A1 sub lineages was plotted using RStudio (v.3.3.4) Venn Diagram package (<https://posit.co/download/rstudio-desktop/>) and the Gene Ontology Biological profile of the selected TFs was plotted with the Shiny GO 0.80 platform (Ge et al., 2020, <http://bioinformatics.sdstate.edu/go/>). **(B)** Pipeline illustrating the TFs binding motifs of the selected TFs, **(C)** The impact of GATA3 on the transcriptional activity of HPV-16 and -18, as obtained from the screening assay. The RLU values presented are the mean of the normalized measurement from two independent experiments conducted in duplicate.

**SUPPLEMENTARY FIGURE 2. Overexpression levels of GATA3 transfected at the HPV positive cell lines. (A)** SiHa, **(B)** CasKi, both HPV-16 positive; and **(C)** SW756 and **(D)** HeLa, both HPV-18 positive.

**SUPPLEMENTARY FIGURE 3. Distribution of predicted GATA3 motifs in the LCR of high-risk HPV types.** We scanned the complete sequence of the LCR for all high-risk HPV types for GATA3 binding motifs with the MoLo Tool algorithm from the HOCOMOCO v.11 database (<https://hocomoco11.autosome.org>, Kulakovskiy et al., 2018). The predicted motifs were further validated using the FIMO algorithm from the MEME Suite 5.5.3 database (https://meme-suite.org/meme/tools/fimo, Charles et al., 2011). Each green square represents a predicted motif, with relative nucleotide positions starting at position 1.

**SUPPLEMENTARY FIGURE 4. GATA3 mRNA expression levels in cervical cancer cell lines.** RT-qPCR analysis of GATA3 transcript levels demonstrated a decrease in GATA3 mRNA in the HPV+ cell lines with the presence of viral oncoproteins E6/E7, except for the HPV-16 positive CaSki cells. The data was normalized to the GAPDH housekeeping gene and quantified using the 2^-ΔΔCt^ method, presented as mean ± SD.

**SUPPLEMENTARY FIGURE 5. GATA3 is predominantly absent in high grade intraepithelial cervical lesions associated with high-risk HPV. (A)** A high-grade HPV-16 positive CIN-3 lesion (indicated by the yellow square) is adjacent to a CIN-1 region (marked with white square). GATA3 expression is absent in the high-grade lesion but remains present in the low-grade tissue. H&E scale = 500 μm, GATA3/DAPI scale = 200 μm. Magnification panels = 50 μm. **(B)** HPV-18 positive sample, where a CIN-1 lesion (shown in the white square) is situated adjacent to a CIN-2 lesion (shown in the yellow square). GATA3 expression can be observed at the CIN-1 site but is absent in the CIN-2 site. H&E scale = 50 μm, GATA3/DAPI = 50 μm. Magnification panels = 10 μm. **(C)** HPV-31 positive sample show normal squamous tissue regions (left panels) with GATA3 present, while high-grade CIN-2 lesions (right panels) display an absence of GATA3. H&E scale = 50 μm and 100 μm, GATA3/DAPI = 50 μm and 100 μm. Magnification panels = 10 μm 3 e 50 μm.
