# Supplementary figures and images for: GATA3 Inhibits the Expression of Viral E6/E7 Genes, and Its Expression Is Compromised During HPV‐Mediated Cervical Carcinogenesis
Source: J Med Virol. 2026 Jun 26;98(7):e71034. doi: 10.1002/jmv.71034 (PMC13306532; doi:10.1002/jmv.71034)

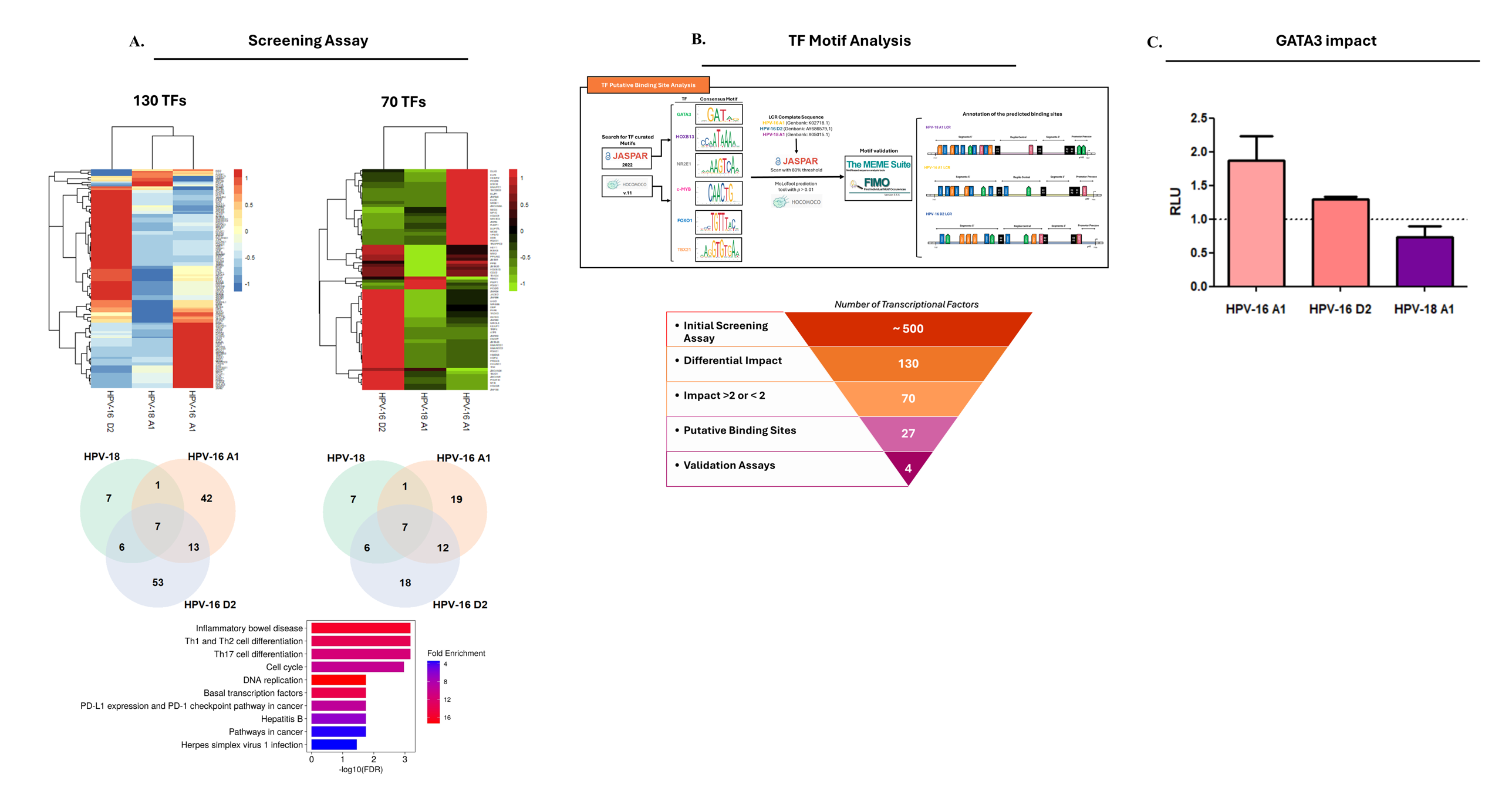

Supplement: Supplementary file 2 — Supporting File 2 [file JMV-98-e71034-s005.tif]

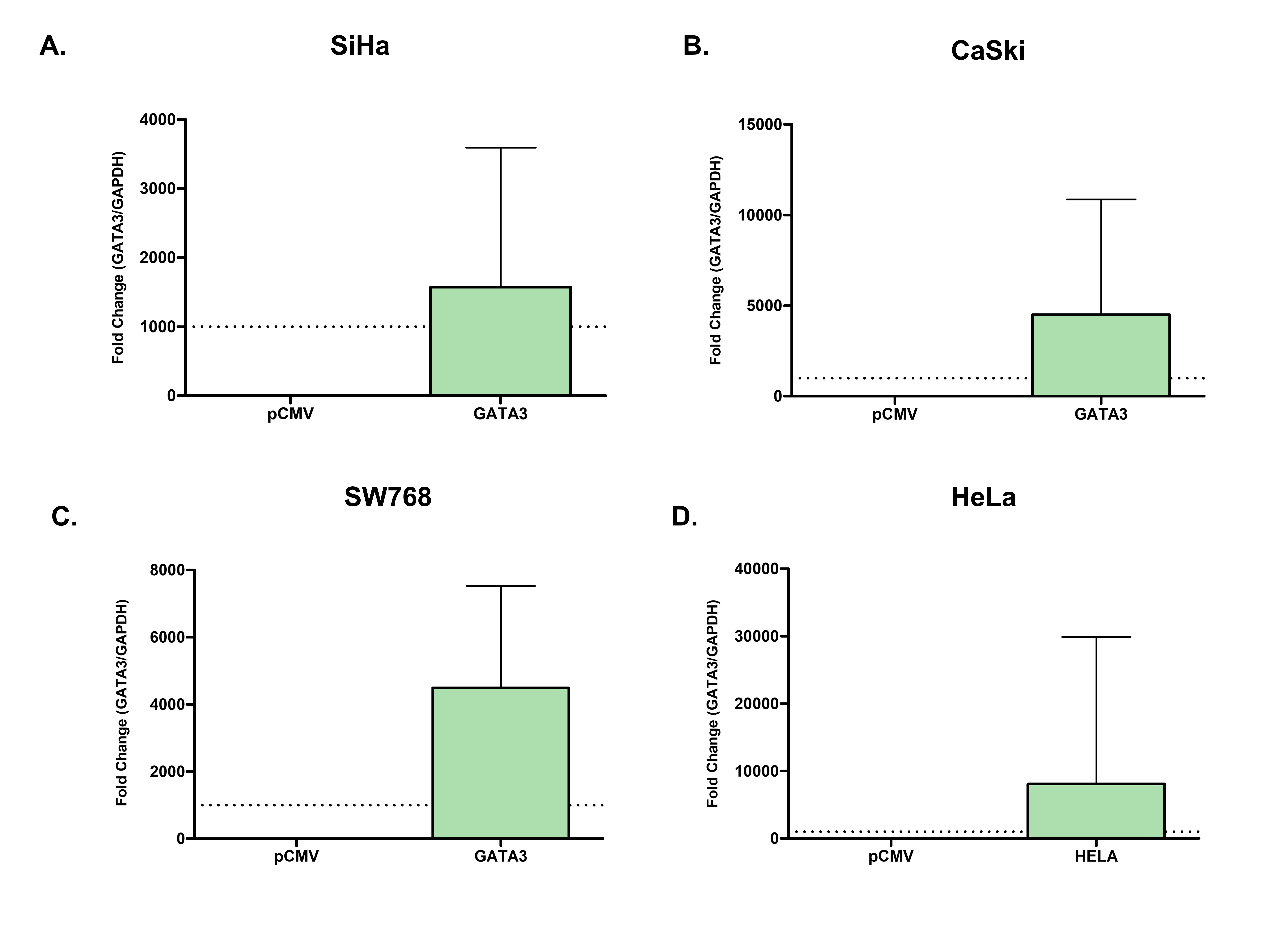

Supplement: Supplementary file 3 — Supporting File 3 [file JMV-98-e71034-s004.tif]

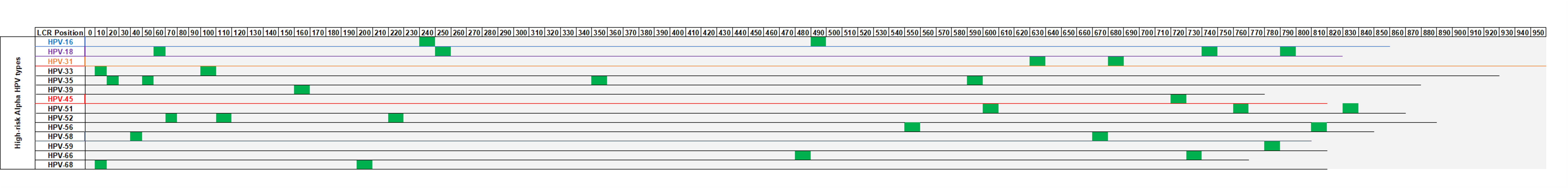

Supplement: Supplementary file 4 — Supporting File 4 [file JMV-98-e71034-s006.tif]

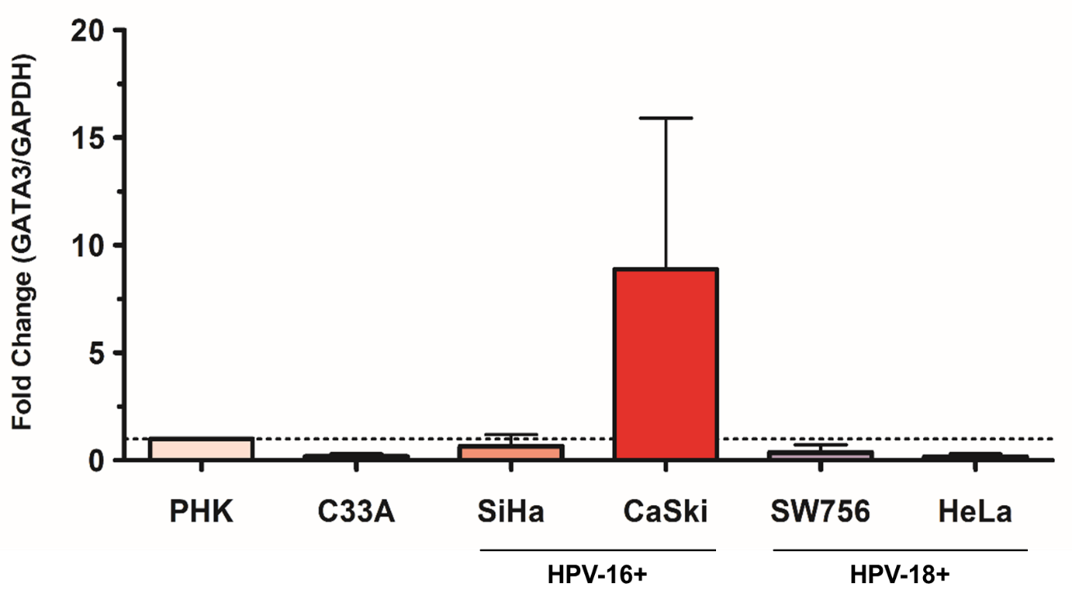

Supplement: Supplementary file 5 — Supporting File 5 [file JMV-98-e71034-s001.tif]

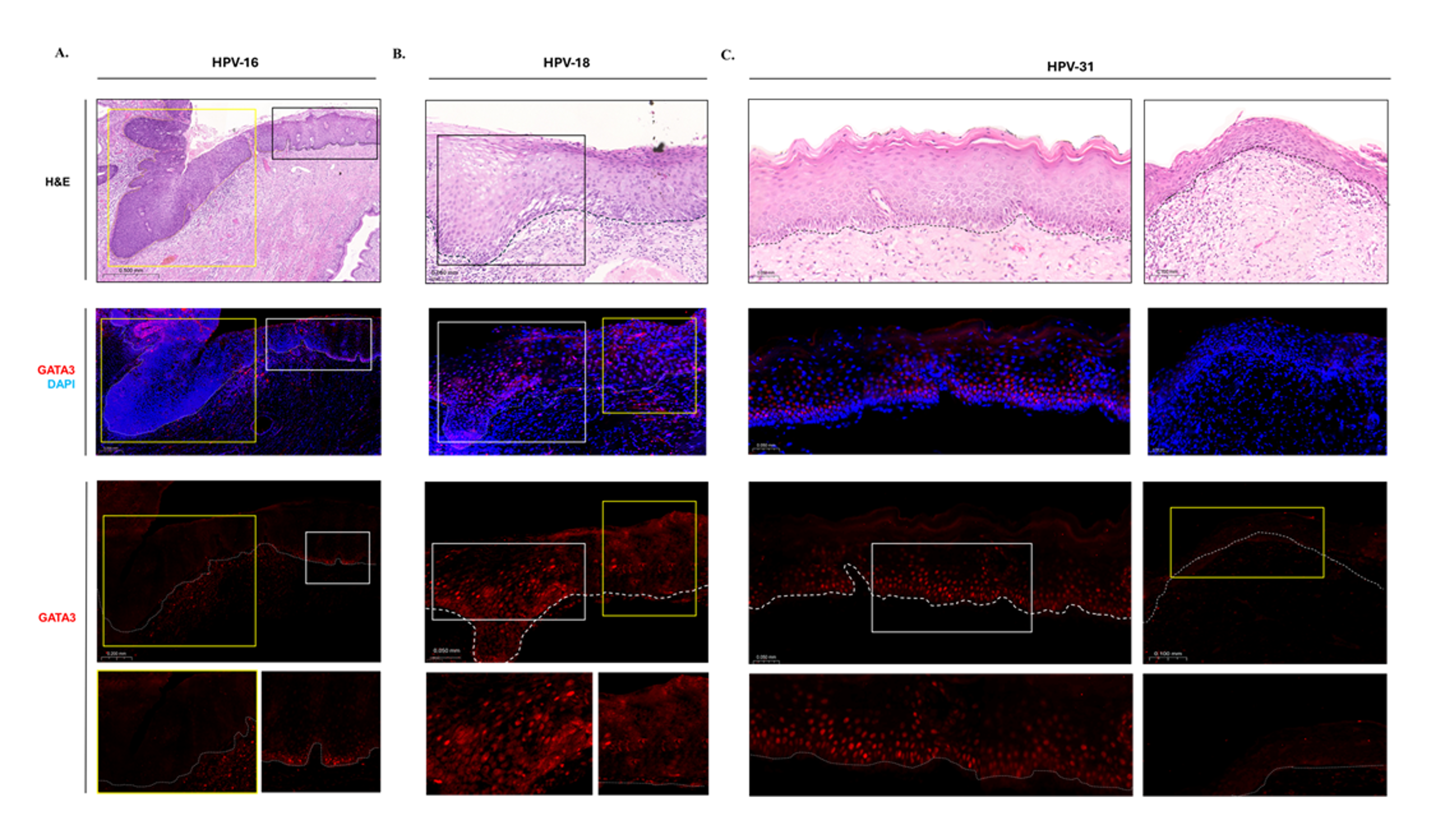

Supplement: Supplementary file 6 — Supporting File 6 [file JMV-98-e71034-s002.tif]
